# Derivation and Characterization of EGFP-Labeled Rabbit Limbal Mesenchymal Stem Cells and Their Potential for Research in Regenerative Ophthalmology

**DOI:** 10.3390/biomedicines9091134

**Published:** 2021-09-01

**Authors:** Julia I. Khorolskaya, Daria A. Perepletchikova, Daniel V. Kachkin, Kirill E. Zhurenkov, Elga I. Alexander-Sinkler, Julia S. Ivanova, Natalia A. Mikhailova, Miralda I. Blinova

**Affiliations:** 1 Institute of Cytology Russian Academy of Science, 194064 St. Petersburg, Russia; dasha_perepletch@mail.ru (D.A.P.); kirill.zhurenkov@incras.ru (K.E.Z.); elga.aleks@gmail.com (E.I.A.-S.); ju.s.ivanova@yandex.ru (J.S.I.); natmik@mail.ru (N.A.M.); mira.blinova@mail.ru (M.I.B.); 2 Laboratory of Amyloid Biology, St. Petersburg State University, 199034 St. Petersburg, Russia; pspdaniel@mail.ru or; 3 Department of Genetics and Biotechnology, St. Petersburg State University, 199034 St. Petersburg, Russia; 4 Sirius University of Science and Technology, 354340 Sochi, Russia

**Keywords:** limbal stem cells, mesenchymal stem cells, limbal mesenchymal stem cells, corneal regeneration, regenerative medicine, mesenchymal-epithelial transition, EGFP-labeled cells

## Abstract

The development of cell-based approaches to the treatment of various cornea pathologies, including limbal stem cell deficiency (LSCD), is an area of current interest in regenerative biomedicine. In this context, the shortage of donor material is urgent, and limbal mesenchymal stem cells (L-MSCs) may become a promising cell source for the development of these novel approaches, being established mainly within the rabbit model. In this study, we obtained and characterized rabbit L-MSCs and modified them with lentiviral transduction to express the green fluorescent protein EGFP (L-MSCs-EGFP). L-MSCs and L-MSCs-EGFP express not only stem cell markers specific for mesenchymal stem cells but also ABCG2, ABCB5, ALDH3A1, PAX6, and p63a specific for limbal epithelial stem cells (LESCs), as well as various cytokeratins (3/12, 15, 19). L-MSCs-EGFP have been proven to differentiate into adipogenic, osteogenic, and chondrogenic directions, as well as to transdifferentiate into epithelial cells. The possibility of using L-MSCs-EGFP to study the biocompatibility of various scaffolds developed to treat corneal pathologies was demonstrated. L-MSCs-EGFP may become a useful tool for studying regenerative processes occurring during the treatment of various corneal pathologies, including LSCD, with the use of cell-based technologies.

## 1. Introduction

Stem cells are mainly responsible for the physiological and reparative regeneration of the tissue. They are present in almost all tissues, including the cornea, where limbal stem cells are a key factor for tissue regeneration. The death of stem cells of the corneal limbus may occur because of external factors–mechanical damage, chemical and thermal burns, genetic diseases, hormonal disorders, etc. [1]. Such damages may lead to different disorders; one of them is limbal stem cell deficiency (LSCD). LSCD is a pathological condition in which damage occurs to the limbal epithelial stem cells (LESCs) or their niche [2].

The limbus is a narrow area between an optically clear cornea and opaque sclera. According to the common conception, the cells of the basal layer of the epithelium- LESCs, in the limbus region, play an important role in the physiological and reparative regeneration of the corneal epithelium [3,4]. If LSCD is extensive, it usually causes corneal epithelial defects, ulceration, and conjunctival overgrowth of the cornea. These changes can lead to neovascularization and corneal opacity, conjunctivalisation, severe inflammation, pain, and visual loss [5]. To date, one of the most common methods of treating these disorders is keratoplasty. Unfortunately, it does not always prove to be an effective method of treatment since donor tissue cannot always replace damaged stem cells. In addition, there is an acute issue of the shortage of donor tissue. Due to a number of clinical trials, cultured human corneal epithelial stem cells from the limbus have been successfully used for corneal restoration [6,7]. However, their use is associated with certain difficulties in isolation and maintaining cells in vitro, as well as with the high cost of these approaches [8].

In the stroma of the limbus region, subjacent to the epithelial basement membrane, corneal stromal stem cells of mesenchymal morphology have been described [9,10]. They are thought to provide sufficient conditions for the normal functioning of LESCs [11,12]. These cells are used in the development of approaches for treating not only corneal stroma pathologies, such as scars, ulcers, and burns but also epithelium loss [13].

Due to the urgent need for alternative sources of stem cells to treat LSCD mesenchymal stem cells (MSCs), the most commonly used adult stem cell in regenerative medicine, from various sources, including the limbal stromal cells, are being investigated to restore the corneal epithelium [14,15,16,17]. In particular, the first clinical study was recently published that showed the effectiveness of the use of bone marrow mesenchymal stem cells (BM-MSCs) for the restoration of the corneal epithelium [8]. Some studies suggest the possibility of transdifferentiation of MSCs of various origins into corneal epithelial cells [18,19,20,21]. However, the exact mechanism of the regenerative effect of MSCs on re-epithelization remains unclear.

In ophthalmological research, rabbits are often used as in vivo models. They have an advantage over other model animals because of their relatively larger eye size compared to rats or mice. However, there are very few studies on rabbit limbal mesenchymal stem cells (L-MSCs) and little data on the characteristics of these cells [22].

In this work, we isolated rabbit L-MSCs, characterized them by markers specific for multipotent stem cells, and compared them with rabbit BM-MSCs. Since there is little information on the participation of L-MSCs in the processes of corneal epithelium restoration, we created a tool that could contribute to a deeper understanding of this issue. We derived a cell culture expressing the green fluorescent protein (EGFP) using lentiviral transduction. This culture will be very useful for future experiments, such as tracking the fate of these cells in tissues after transplantation. We also compared the populations of the obtained L-MSCs and L-MSCs-EGFP. It has been shown that both cultures contain stem cell markers specific for LESCs and various cytokeratins. We demonstrated that cells cultivated under conditions that simulate the physiological environment of the epithelium could transdifferentiate into epithelial-like cells. In addition, the use of L-MSCs-EGFP can be very beneficial in the research and development of various scaffolds for creating tissue-engineered corneal substitutes.

## 2. Materials and Methods

### 2.1. Cell Cultures

Rabbit L-MSCs were isolated from corneo-scleral rims of a male Chinchilla rabbit obtained during the formation of a model of limbal stem cell deficiency. The surgical procedure included careful dissection of a sample of limbal tissue with 0.5 mm depth, originating 3 mm behind the limbus and extending into corneal stromal tissue at the limbus. Animals were housed and treated according to Animal Welfare Assurance of INC RAS (IN F18-00380, 2017–2022). Limbal tissues were de-epithelialized using Dispase II (Roche, Basel, Switzerland) at a concentration of 2.4 U/mL overnight at +4 °C and next day at +37 °C for 30 min. Fragments of de-epithelized tissue were placed on culture dishes and covered with coverslips. The primary culture was incubated in DMEM/F12 medium (Gibco, Waltham, MA, USA) supplemented with 10% fetal bovine serum (FBS) (Gibco, Waltham, MA, USA), 1000 U/mL Pen/Str (Gibco, Waltham, MA, USA), 0.5 ng/mL amphotericin B (Gibco, Waltham, MA, USA). After reaching 80–90% confluency, cultures were harvested with 0.25% trypsin-EDTA (Gibco, Waltham, MA, USA) and cultured for 10 passages.

Rabbit BM-MSCs were kindly provided by Dr. Alexandrova S.A. from the Cell Technologies Center of the Institute of Cytology, Russian Academy of Sciences.

All cultures were maintained in DMEM/F12 medium supplemented with 10% FBS, 1000 U/mL Pen/Str in 5% CO_2_ in a humidified incubator at 37 °C.

### 2.2. Obtaining of Rabbit L-MSCs with Overexpression of Green Fluorescence Protein (rbL-MSC-EGFP)

To make a cell culture of rabbit L-MSCs labeled with a green fluorescent protein EGFP, a lentiviral vector LV-CMV-EGFP Hygro (656-4) was generated, as previously described [23]. The basic plasmid pLenti-CMV-EGFP Hygro (656-4) [24] was used for the production of the second-generation lentivirus assembly system. All stages of obtaining lentiviral vectors were performed with appropriate biosafety protection recommended for studies using and obtaining lentiviral vectors [25].

Rabbit L-MSCs at passage 3 were transduced with the lentivirus LV-CMV-EGFP Hygro (656-4) in Opti-MEM medium (Gibco, Waltham, MA, USA) using polybrene (Sigma-Aldrich, St. Louis, MO, USA) at a concentration of 8 μg/mL. Lentivirus LV-CMV-EGFP Hygro (656-4) was added in an amount of 5 MOI to rabbit L-MSCs and incubated in the presence of the virus for 20 h. After 72 h of transduction, cells expressing EGFP were sorted using an S3e cell sorter (BioRad, Laboratories, Hercules, CA, USA).

Evaluation of transduction efficiency was based on the analysis of EGFP fluorescence intensity measured by CytoFLEX flow cytometer (Beckman Coulter, Brea, CA, USA) (488 nm laser).

### 2.3. Cell Proliferation Assay

To characterize the proliferative activity of L-MSCs and L-MSCs-EGFP at 8 passage, the doubling time (D_t_) was estimated, and the growth curves of cell populations were made. To measure the mean time of cell population doubling in each cell lineage, three repeats were analyzed daily, counting the number of cells for 5 days (120 h).

24 h before the start of the first measurement, 10.000 cells per well were plated on a 12-well plate in DMEM/F12 medium supplemented with 10% FBS; and 1000 U/mL Pen/Str. Cell counting was performed every 24 h for 5 days using a CytoFLEX flow cytometer.

MDT was calculated by the formula:

(1) Dt=(t2−t1)ln2ln(M2M1),

where t_1_ and t_2_—are the times of cell counting after the start of the assay, M_2_ number of cells at time t_2_, M_1_ number of cells at time t_1,_ and D_t_ time of cell doubling [26]. Data were presented as the mean ± standard deviation.

### 2.4. Cell Surface Marker Analysis

Rabbit BM-MSCs, L-MSCs, and L-MSCs-EGFP were characterized for mesenchymal (CD90, CD105, CD44, CD73) and hematopoietic (CD34, CD45) markers by flow cytometry using antibodies conjugated with Phycoerythrin (PE) (Table 1).

Cells, harvested with 0.25% trypsin-EDTA solution, were suspended in complete DMEM/F12 medium, centrifuged, and resuspended in phosphate-buffered saline (PBS) to reach the concentration of 1 × 10^6^ cells/mL. A total of 30,000 cells for each sample were incubated with the appropriate primary conjugated antibodies for 60 min in the dark at room temperature, then were diluted with PBS (1:10) and analyzed using the CytoFLEX Flow Cytometer (561 nm laser). Isotype controls were used as negative controls.

### 2.5. Immunofluorescence

The expression of selected markers was confirmed by immunocytochemistry using antibodies listed in Table 2. Rabbit L-MSCs and L-MSCs-EGFP P6 were seeded on coverslips in 24-well culture plates and cultured to confluency. They were then were fixed with 1% paraformaldehyde (PFA) (Sigma-Aldrich, St. Louis, MO, USA) for 20 min, permeabilized with 0.1% Triton X-100, and blocked with 10% FBS and 1% BSA (Thermo Fisher Scientific, Waltham, MA, USA) solution. Cells weren’t treated with Triton X-100 when processed with antibodies to E-cadherin. Then, cells were incubated with primary antibodies overnight at +4 °C. After several washes, the proper secondary antibody was added for 60 min at room temperature. The nuclei were counterstained with DAPI (1 μg/mL) (Thermo Fisher Scientific, Waltham, MA, USA). Cells were observed using an OLYMPUS FV3000 confocal microscope (Olympus, Center Valley, PA, USA). ImageJ (v.2.1 software) was used to process and analyze the obtained images.

### 2.6. In Vitro Multilineage Differentiation

The ability of L-MSCs-EGFP to differentiate into adipocytes, osteocytes, and chondrocytes was determined using StemPro Osteogenesis, Adipogenesis, and Chondrogenesis Differentiation Kits (Thermo Fisher Scientific, Waltham, MA, USA) following the manufacturer’s instructions. For adipogenic and osteogenic differentiation L-MSCs-EGFP P6 were seeded at a density of 10,000 cells/cm^2^ and cultured for 21 days. At the end of the cultivation period, the cultures were fixed with 4% PFA and stained with specific dyes.

To assess adipogenic differentiation of L-MSCs-EGFP, cells were stained with Nile Red (Invitrogen, Carlsbad, CA, USA). A stock solution of Nile Red was made at a concentration of 10 mg/mL on DMSO. Nile Red Staining Solution (10 μg/mL in PBS) was used for staining. The staining of the fixed cells was carried out for 10 min at 37 °C. The nuclei were stained with DAPI (1 μg/mL). Visualization was performed using an OLYMPUS FV3000 confocal microscope.

The presence of calcium salts in the extracellular matrix of the L-MSCs-EGFP culture was used as a marker of osteogenic differentiation. Calcium salts were detected by staining with Alizarin Red S Staining Kit (ScienCell, Carlsbad, CA, USA) for 15 min at room temperature. Also, osteogenic differentiation of L-MSCs-EGFP was demonstrated by alkaline phosphatase staining using BCIP/NBT Substrate Solution (Thermo Fisher Scientific, Waltham, MA, USA) for 2 h at 37 °C. Then the stained cells were observed under the microscope Leica DM2500 (Leica Microsystems GmbH, Wetzlar, Germany).

For chondrogenic differentiation, cell suspension of L-MSCs-EGFP P6 of 1 × 10^6^ cells/mL were pelleted, and micromass was generated in the centrifuge tube. Micromass of L-MSCs-EGFP was cultured for 14 days in 5% CO_2_ in a humidified incubator at 37 °C. At the end of the induction period, the micromass was transferred onto a glass slide and dispersed. A 0.1% Safranin O solution (Lenreaktiv, St. Petersburg, Russia) was used to detect chondrogenic differentiation. The smears were fixed with 4% PFA. Staining was carried out for 30 min at room temperature, followed by washing with distilled water. Then the stained cells were observed under the microscope Leica DM2500 (Leica Microsystems GmbH, Wetzlar, Germany).

### 2.7. Epithelial Transdifferentiation

L-MSCs-EGFP were seeded at a density of 1 × 104 cells/cm^2^ into cell culture inserts (Jet Biofil, Guangzhou, China) and cultured for 14 days in a CnT-30 medium. The epithelial corneal medium was changed only in the outer well of the culture plate (CellnTec Advanced Cell Systems, Bern, Switzerland). Control cells were maintained in DMEM/F12 supplemented with 10% FBS and cultured alongside. The culture medium was changed every 3 days. At the end of the induction, the cells on the inserts were fixed with 1% PFA solution and stained with antibodies for CK 15, E-cadherin, and Vimentin, as described above (Immunofluorescence section). To visualize actin microfilaments, Phalloidin-TRIC staining (Invitrogen, Carlsbad, CA, USA) was performed. The nuclei were stained with DAPI (1 μg/mL). Cells were observed using an OLYMPUS FV3000 confocal microscope.

Quantitative image analysis of images was performed using ImageJ (v.2.1 software). Data were presented as the mean ± standard deviation.

### 2.8. Quantitative RT-PCR Analysis

To assess the level of expression of several genes regulating the processes of epithelial-mesenchymal transition—SNAI2, TWIST1, and ACTA2, the RT-qPCR assay was performed in L-MSCs-EGFP. Total RNA was extracted from L-MSCs-EGFP cultured in CnT30 culture medium on tissue culture plate inserts and L-MSCs-EGFP cultured on a culture plate in complete DMEM/F12 medium as a control. To isolate RNA, the ExtractRNA reagent (Evrogen, Moscow, Russia) was used according to the manufacturer’s protocol. The quality of the isolated RNA was verified by using a 1% agarose gel electrophoresis to assess the integrity of total RNA and Nanodrop 1000 spectrophotometer (Thermo Scientific, Waltham, MA, USA) to ensure the 260/280 ratio was within the 1.8–2.0 range. Synthesis of cDNA from isolated total RNA (500 ng) was done using the MMLV RT kit (Evrogen, Moscow, Russia) using random (dN)_10_ primers, according to the manufacturer’s protocol, in a T100 Thermal Cycler (Bio-Rad Laboratories, Hercules, CA, USA). The quantitative polymerase chain reactions (PCR) were carried out using qPCRmix-HS SYBR + LowROX (Evrogen, Moscow, Russia), according to the manufacturer’s protocol, in a LightCycler^®^ 96 (Roche, Basel, Switzerland), with the following 40 × three-step cycle: 10 s at 95 °C, 30 s at 60 °C, and 15 s at 72 °C.

The transcription levels of ACTA2, SNAI2 (Slug), and TWIST1 using specific primers (Table 3) were calculated by the delta-delta Ct (ΔΔCt) method and normalized to the expression of the HPRT1 housekeeping gene. Data were represented as gene expression relative to that of control cells (LSC-EGFP on DMEM/F12) from three independent experiments (*n* = 3).

### 2.9. Lifetime Evaluation of Scaffolds Biocompatibility

To analyze the morphology of L-MSCs-EGFP during cultivation on different opaque scaffolds, a decellularized amniotic membrane (AM), collagen hydrogel, and Poly(D, L-lactide)/PEG (PLA) film were used.

The amniotic membrane was decellularized and prepared according to the previously described technique [27].

PLA film was provided by the colleges of the Cell Technology Center and prepared according to the previously described method [28].

Cells on the surface of AM and PLA films were seeded (1 × 10^4^ cells/cm^2^) and cultured for 7 days in DMEM/F12 supplemented with 10% FBS.

Collagen hydrogel was prepared as previously described [29] from type I collagen, developed at the Institute of Cytology, Russian Academy of Sciences [30]. Type I collagen was mixed with 10× medium 199 (Gibco, Waltham, MA, USA) and sterile 0.34N NaOH solution (Sigma-Aldrich, St. Louis, MO, USA). The mixture was supplemented with the cell suspension of L-MSCs-EGFP (1 × 10^5^ cells/mL). The final concentration of collagen was 2 mg/mL. The suspension was incubated in a CO_2_ incubator at 37 °C for 30 min until full collagen polymerization. Then DMEM/F12 supplemented with 10% FBS was added, and cells were cultured in 3D condition for 7 days.

The morphology of L-MSCs-EGFP cultured on scaffolds was observed during the entire cultivation period using The ZOE Fluorescent Cell Imager (BioRad Laboratories, Hercules, CA, USA) in the GFP channel.

### 2.10. Statistical Analysis

In all experiments, error bars represent the standard deviation (S.D.) of the mean, analysed a priori for homogeneity of variance. For statistical analysis of gene expression, replicates from each independent experiment were confirmed to follow a Gaussian distribution, and differences between groups were determined using two-way analysis of variance (ANOVA) followed by Bonferroni’s multiple comparison post hoc test. Significance between groups was established for *p* < 0.001, and *p* < 0.0001, and with a 95% confidence interval. The average ± S.D. Statistical analysis for growth curves data was performed using simple linear regression analysis with a 95% confidence interval. All statistical calculations and graphs plotting were performed using Prism 9.0 (GraphPad Software, San Diego, CA, USA).

## 3. Results

### 3.1. Establishment of L-MSCs Culture of Rabbit

**Figure 1:**
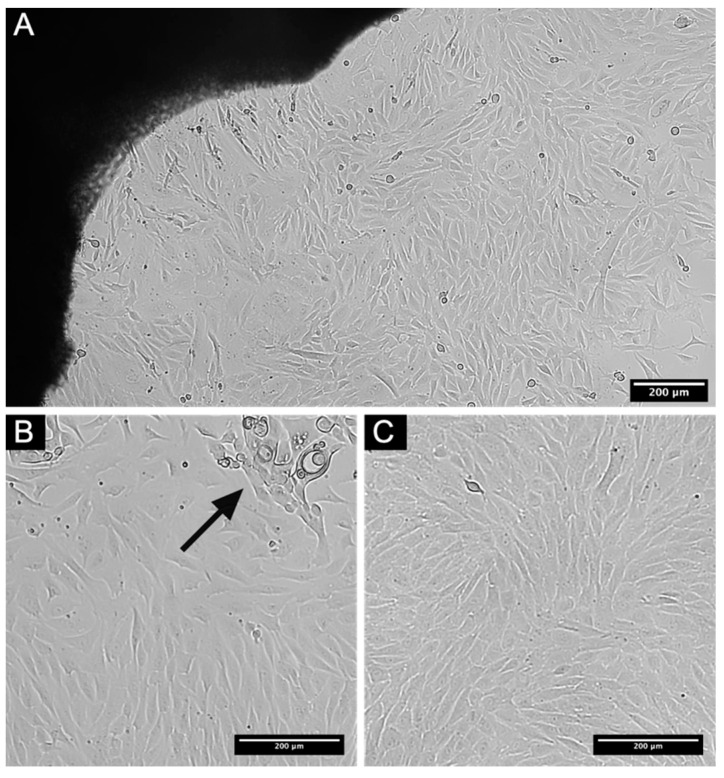
Phase-contrast microscopy of cell culture isolated from rabbit corneo-scleral rims. (**A**) Primary culture isolated from rabbit limbus. Cells appear elongated with single nucleus. (**B**) 21 days of cultivation of primary culture. Cell monolayer was formed. Arrow marks cells of epithelial morphology. (**C**) Established homogenous cell culture after 3rd passage. Scale bars: (**A**–**C**). 200 micrometers (μm).

De-epithelized rabbit limbus fragments after treatment with Dispase II were used to isolate L-MSCs by the migration method. A heterogeneous culture represented mainly by cells with mesenchymal morphology was isolated from the limbus explants. Under a phase-contrast microscope, the cells appeared elongated with a single nucleus (Figure 1A). After 21 days of cultivation of the primary culture, a cell monolayer was formed. Prior to the first passage, heterogeneous cell culture was observed, represented by both cells with epithelial morphology and mesenchymal cells of the limbal stroma (Figure 1B). After several passages, the cell population became more homogeneous. By the 3rd passage, no cells with typical epithelial morphology remained in culture (Figure 1C).

### 3.2. Obtaining of Rabbit L-MSCs-EGFP Culture

**Figure 2:**
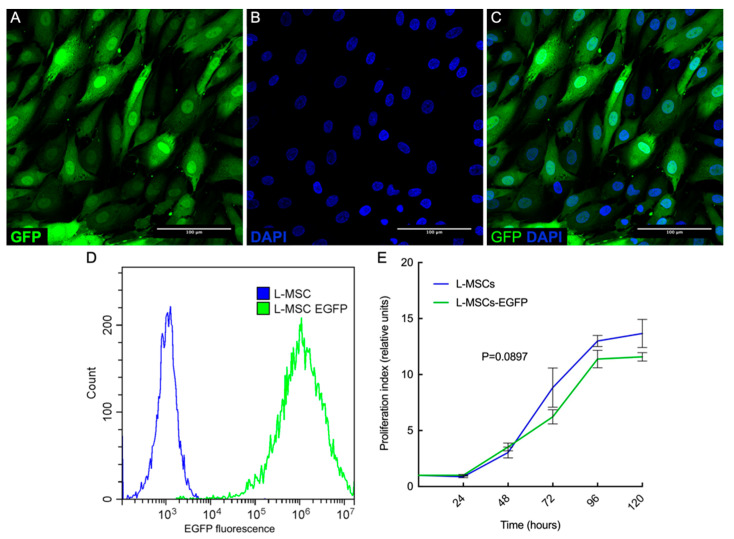
Analysis of L-MSCs with overexpression of green fluorescent protein (L-MSCs-EGFP). (**A**–**C**)—confocal microscopy of fixed L-MSCs-EGFP stained with DAPI (Scale bar—100 μm). (**A**) GFP channel. (**B**) DAPI channel. (**C**) Merge of two channels (GFP + DAPI). (**D**) Flow cytometric analysis of L-MSCs and the population of L-MSCs-EGFP after cell sorting. In GFP-channel the high fluorescence intensity of EGFP protein in the entire population of L-MSCs-EGFP was shown. The blue curve corresponds to L-MSCs, and the green curve corresponds to L-MSCs-EGFP. Curves describe the intensity of the EGFP fluorescence signal in the cell populations. (**E**) Cell proliferation assay of rabbit L-MSCs and L-MSCs-EGFP. There were no significant differences in cell proliferation rates (*p* < 0.09).

To make L-MSCs-EGFP, a lentiviral vector encoding the fluorescent protein EGFP sequence controlled by the constitutive CMV promoter was preliminarily obtained [23]. This lentiviral vector was used to transduce L-MSCs isolated from the rabbit limbus. Three days after infection, transduction efficiency was determined by flow cytometry and was over 80%. Cells expressing EGFP were sorted using an S3e cell sorter (BioRad, USA). The resulting culture stably expresses the green fluorescent protein EGFP (L-MSCs-EGFP), which is evenly spread over the cytoplasm and nucleus (Figure 2A–C). Flow cytometric analysis of transduction efficiency showed the high fluorescence intensity of EGFP protein in the entire population of L-MSCs-EGFP (Figure 2D).

### 3.3. Characterization and Comparison of Rabbit L-MSCs and L-MSCs-EGFP

To assess whether, after viral transduction, serious changes in cells occurred in the culture of L-MSCs-EGFP, we compared these two cultures in terms of proliferative activity, the expression profile of surface antigens, some differentiation, and stem cell markers.

#### 3.3.1. Comparison of Cell Proliferation Rates

Growth curves were plotted for L-MSCs and L-MSCs-EGFP at passage 8 (Figure 2E). The duration of the logarithmic phase for both cultures was 72 h. By plotting the growth curve of the obtained cultures, as well as calculating the average doubling time of these populations, it was shown that after transduction, the proliferative activity of L-MSCs-EGFP was slightly lower than in cells that did not undergo genetic changes (26 against 27 h). However, it retains a high level of proliferative activity and can be multiplied in vitro in sufficient quantities not only for experimental studies but also for biobanking.

#### 3.3.2. Cell Surface Marker Analysis

L-MSCs and L-MSCs-EGFP, as well as rabbit BM-MSCs, were characterized at passage 8 for mesenchymal (CD90, CD105, CD44, CD73) and hematopoietic (CD34, CD45) markers by flow cytometry. Table 4 summarizes the surface marker expression profile. Due to the lack of antibodies produced against rabbit cell surface antigens, the panel was supplemented with antibodies against mice or human antigens to observe any cross-reactivity.

CD90 and CD44, specific for MSCs, were expressed on all three types of cells with fairly high efficiency. However, the L-MSCs and L-MSCs-EGFP cultures were found to be more heterogeneous in terms of the CD105 expression profile. The analysis revealed low levels of reactivity with antibodies against CD73 for all three cell types, which should normally be present on the MSCs. This may be due to the use of non-rabbit-specific antibodies. All three types of cells have shown no expression of hematopoietic markers (CD34 and CD45).

#### 3.3.3. L-MSCs and L-MSCs-EGFP Characterization and Comparison by Immunocytochemistry

**Figure 3:**
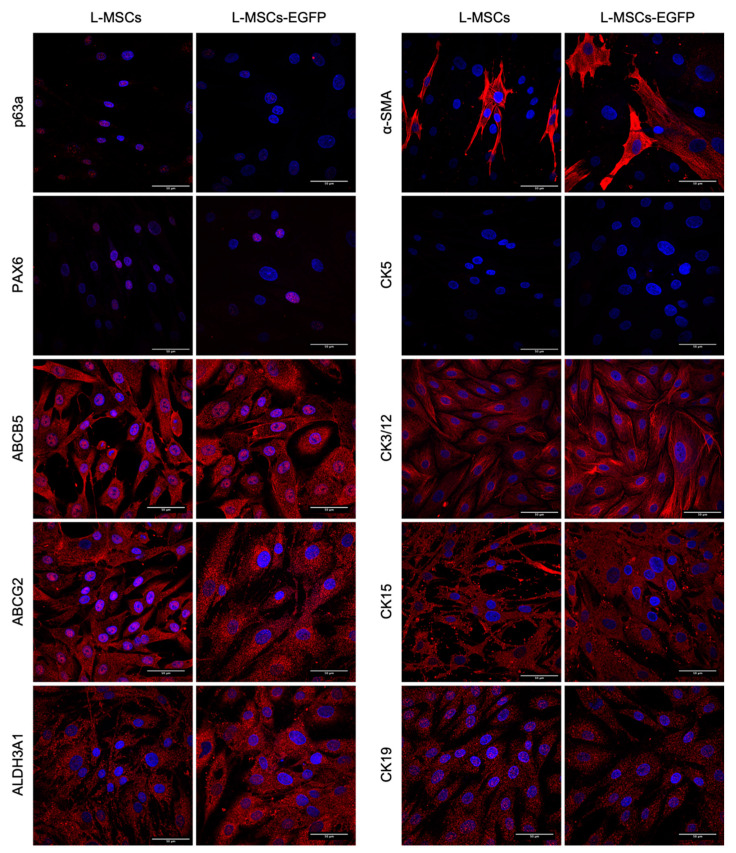
Confocal images of immunocytochemistry assay of L-MSCs and L-MSCs-EGFP. Cells were stained with primary antibodies against stem cells markers (p63α, PAX6, ABCB5, ABCG2, and ALDH3A1), cells differentiation marker (α-SMA), and cytokeratins (CK5, CK3/12, CK15 and CK19) and AlexaFluor conjugated secondary antibodies. The nuclei were counterstained with DAPI. Scale bars—50 micrometers (μm).

The obtained cultures of L-MSCs and L-MSCs-EGFP at the 6th passage were characterized by the stem cell and differentiation markers, which are used to describe stem cells of the stroma and epithelium of the limbus (Figure 3). As limbal stem-cell-associated markers, transcriptional factors p63α and PAX6 were used. In both compared cultures, a signal in the nuclei was observed for p63α and PAX6. However, the signal was not very strong. Both L-MSCs and L-MSCs-EGFP showed a strong positive response to ABCB5, ABCG2 markers, that are used for both epithelial and stroma limbal stem cells. The signal was observed both in the cytoplasm and in the nuclei. ALDH3A1 also was present in the cytoplasm of both cell types.

The expression of αSMA was observed only in a part of the population, which may indicate its heterogeneity. αSMA forms fibrillar structures in the cytoplasm of individual cells.

Also, CK3/12, CK15, and CK19 have been found in the L-MSCs and L-MSCs-EGFP cytoplasm. CK3/12 formed well-defined fibrillar structures; CK15 and CK19, on the contrary, did not form fibrillar structures but were distributed over the cytoplasm of cells. For CK19, nuclear localization was also observed in some cells. Both cultures were negative for CK5.

Thus, we did not find any difference in the expression of the selected markers after transduction with the lentiviral vector.

### 3.4. Ability of L-MSCs-EGFP to Multilineage Differentiation

L-MSCs-EGFP were differentiated in vitro using adipogenic, osteogenic, and chondrogenic induction media. Cells cultured alongside under standard conditions in complete DMEM/F12 medium were used as a control.

**Figure 4:**
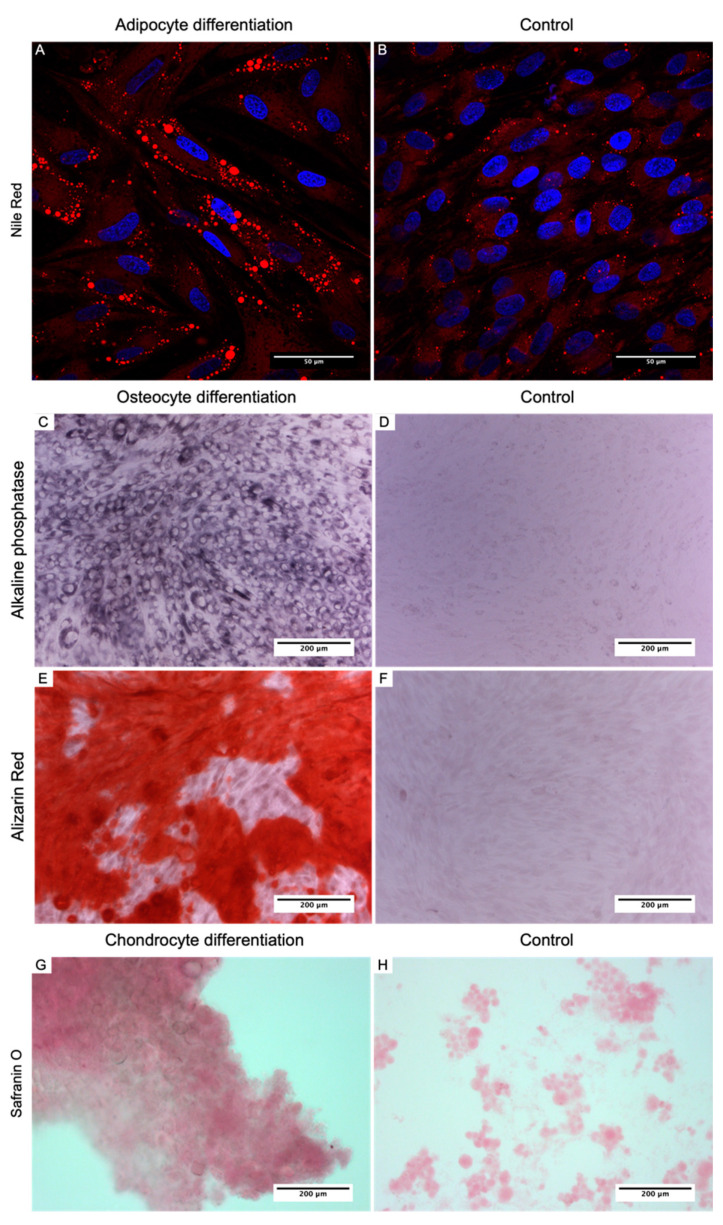
(**A**,**B**)—confocal images of L-MSCs-EGFP stained with Nile Red. The nuclei were counterstained with DAPI. Scale bar 50 μm. (**A**) L-MSCs-EGFP after 3 weeks induction in adipogenic media. (**B**) L-MSCs-EGFP control cells. (**C**,**D**) light microscopy of L-MSCs-EGFP stained for alkaline phosphatase (Scale bar 200 μm). (**C**) L-MSCs-EGFP after 3 weeks of induction in chondrogenic media. (**D**) control cells. (**E**,**F**) light microscopy of L-MSCs-EGFP stained with Alizarin Red (Scale bar 200 μm). (**E**) L-MSCs-EGFP after 3 weeks of induction in chondrogenic media. (**F**) control cells. (**G**,**H**) light microscopy of L-MSCs-EGFP micromasses stained with Safranin O (Scale bar 200 μm). (**G**) micromass of L-MSCs-EGFP after 2 weeks of induction in chondrogenic media. (**H**) control cells.

Three weeks after the adipogenic induction, the Nile Red staining showed the formation of large lipid drops in the cytoplasm of differentiated cells (Figure 4A,B). That indicated the differentiation of L-MSCs-EGFP into adipocyte-like cells.

L-MSCs-EGFP induced to osteogenic differentiation stained for alkaline phosphatase after three weeks of induction (Figure 4C,D) and stained with alizarin red for calcium deposits (Figure 4E,F). The alkaline phosphatase activity and the presence of calcium deposits from the alizarin red staining were clearly observed after differentiation, which was not present in the undifferentiated cells.

During the chondrogenic differentiation of L-MSCs-EGFP, the formed micromass cultured in a differentiation medium for 2 weeks was elastic and separated tightly into parts during smear preparation. The L-MSCs-EGFP cultured in a standard growth medium did not form a micromass and disintegrated during smear preparation. Staining with Safranin O showed a greater number of proteoglycans in the micromass formed by L-MSCs-EGFP after differentiation in a chondrogenic medium than in control culture (Figure 4G,H).

### 3.5. Direct Epithelial Transdifferentiation of L-MSCs-EGFP

L-MSCs-EGFP were cultured in tissue culture inserts in a CnT30 medium designed for corneal differentiation of pluripotent stem cells. Cells on these inserts get nutrients through the membrane at the bottom of the insert. Such air-lifting conditions can provide an additional stimulus for differentiation in the epithelial direction. L-MSCs-EGFP were cultured in this system for 14 days. Cells cultured on glass in a complete DMEM/F12 medium were used as a control.

**Figure 5:**
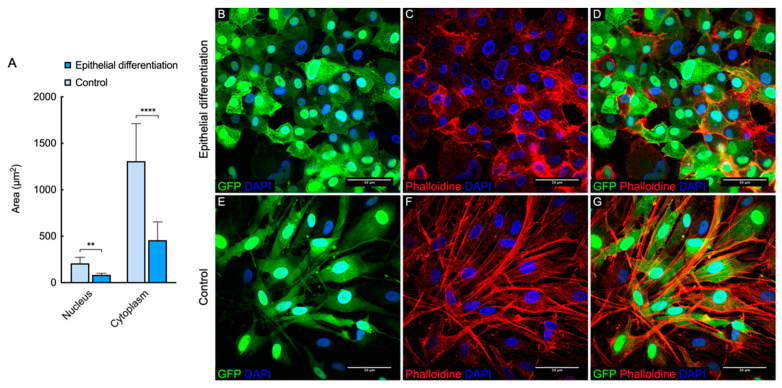
Confocal images of the actin cytoskeleton of L-MSCs-EGFP stained with DAPI Phalloidin-TRIC after induction in epithelial medium (**A**–**C**) and control L-MSCs-EGFP (**D**–**F**). (**A**,**D**) DAPI and GFP channels. (**B**,**E**) DAPI and TRITC channel. (**C**,**F**) Merge of three channels (DAPI, GFP, TRITC). The scale bar on all images 50 micrometers (μm). (**G**) The plot illustrates the change in the size of cells and nuclei during epithelial differentiation. Data represented as average ± S.D. from three independent experiments (*n* = 3; ** and **** corresponds to *p* < 0.001 and 0.0001 after two-way ANOVA, respectively).

It has been shown that control cells have a typical MSCs-like spindle-shaped morphology. When cultured on the insert, L-MSCs-EGFP were smaller, had a hexagonal morphology, and the size of the nuclei became smaller (Figure 5A).

Phalloidin staining showed changes in the structure of the actin cytoskeleton. It was shown that after 14 days of differentiation, actin acquired a predominantly cortical localization, while in the control, numerous well-defined stress fibrils were observed in the cytoplasm of the cells (Figure 5B–G).

**Figure 6:**
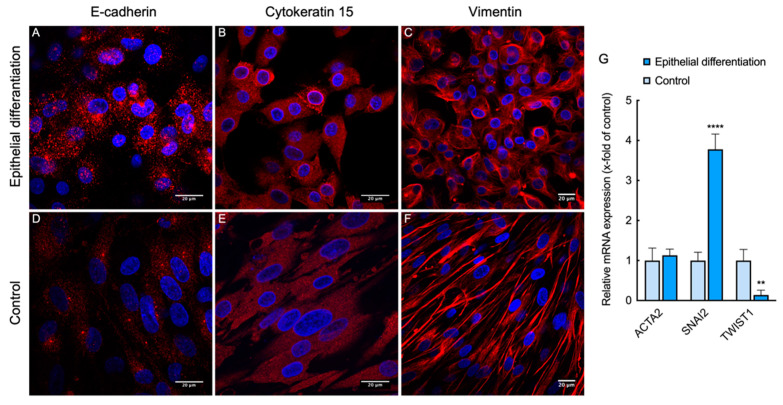
Confocal images of immunocytochemistry assay of L-MSCs-EGFP after 14 days of epithelial differentiation. Cells were stained with primary antibodies against marker associated with cell junctions—E-cadherin (**A**,**D**), and intermediate filaments—cytokeratin 15 (**B**,**E**) and vimentin (**C**,**F**). (**A**–**C**) demonstrate L-MSCs-EGFP after induction in epithelial differentiation medium. (**D**–**F**) demonstrate control L-MSCs-EGFP. Scale bars—20 micrometers (μm). (**G**). Comparative gene expression analysis of ACTA2, SNAI2, and TWIST1 in L-MSCs-EGFP after 14 days of epithelial differentiation. The plot shows the expression of mRNA in studied cells relative to the housekeeping gene HPRT1. Data represented as average ± S.D. from three independent experiments (*n* = 3; ** and **** corresponds to *p* < 0.001 and 0.0001 after two-way ANOVA, respectively).

Epithelial differentiation of L-MSCs-EGFP was analyzed by immunofluorescent staining against epithelial markers CK 15, E-cadherin, as well as the mesenchymal cell marker vimentin (Figure 6A–F). Although the expression of all these markers was observed in both differentiated and undifferentiated L-MSCs-EGFP, some changes were observed in their expression pattern. Expression of E-cadherin was more pronounced in the cells after induction. A well-defined CK15-ring around the nucleus was detected in a number of differentiating cells, while such structures were not formed in the control cells. When staining with antibodies against Vimentin, long straight fibrils were noted in the control, while after induction of epithelial differentiation, Vimentin was detected as a dense network of thin fibrils.

Quantitative analysis of gene expression was performed on L-MSCs-EGFP after cultivation in corneal epithelial differentiation medium on tissue culture inserts (Figure 6G). L-MSCs-EGFP cultured on plastic in complete DMEM/F12 medium for 14 days was used as control cells. RT-qPCR showed a significant increase in SNAI2 expression by 3.8 times and a decrease in TWIST1 expression in the cells cultured with air-lifting. Expression of the ACTA2 gene did not change.

ACTA2 is one of the mesenchymal markers of epithelial-mesenchymal transition (EMT). As well as SNAI2 and TWIST1, these are transcription factors capable of inducing EMT by regulating the expression of E-cadherin. Based on the immunofluorescence and RT-PCR data, we suggest that the cells cultured in the described conditions are not fully differentiated but are in the process of mesenchymal-epithelial transition.

### 3.6. Lifetime Evaluation of Biomedical Scaffolds Biocompatibility with L-MSCs-EGFP

We assume that the obtained genetically modified culture of L-MSCs-EGFP can become a convenient tool for not only in vivo but also in vitro research. During the development of tissue-engineered constructs for the restoration of the corneal stroma or epithelium, special attention is paid to the biocompatibility of the cell component and the scaffold. However, it is not always possible to assess the viability and morphology of cells cultured on scaffolds because of their low transparency. Live-cell microscopy of L-MSCs-EGFP was performed using ZOE Fluorescent Cell Imager. The presence of the EGFP protein evenly distributed over the cell cytoplasm makes it possible to evaluate any changes in the morphology of these cells, as well as to carry out quantitative analysis.

**Figure 7:**
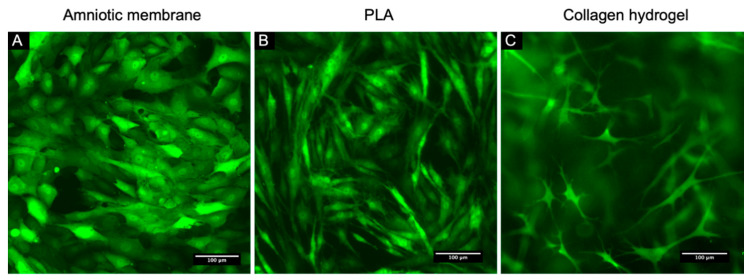
Live-cell fluorescence microscopy of L-MSCs-EGFP on different scaffolds after 7 days of cultivation. (**A**). L-MSCs-EGFP on decellularized AM. (**B**). L-MSCs-EGFP on PLA scaffold. (**C**). L-MSCs-EGFP on collagen hydrogel. Scale bars: (**A**–**C**) 100 micrometers (μm).

It was shown that during the culturing of L-MSCs-EGFP on the epithelial side of the decellularized amniotic membrane, the cells adopted more epithelial-like morphology, the cells on the PLA film had a typical MSCs-like morphology, and inside the collagen hydrogel, they formed a 3D network with a large number of branches (Figure 7).

## 4. Discussion

The first works describing MSCs-like limbal stem cells appeared at the beginning of the 21st century [9,10,12,31]. Today, various ways to isolate stem cells from the limbal stroma have been described. In various publications, authors name these cultures differently: limbal stem cells [32], limbal mesenchymal cells [12], corneal stromal stem cells [9], limbal niche cells [33], limbal biopsy–derived stromal cells (LBSCs) [34]. There are also difficulties with the characteristics of this population. Morphologically, these cells look similar to BM-MSCs under the phase-contrast microscope. Some authors compare them with MSCs of various origins [12,20,35]. According to the minimum criteria established by the International Society for Cellular Therapy, MSCs should match the following criteria: (1) be adhesive to plastic; (2) differentiate in osteogenic, chondrogenic, and adipogenic directions; (3) present specific surface antigens such as CD73, CD90, CD105 and should not express CD34 and CD45 markers [36]. It has also been shown that human L-MSCs can differentiate in osteogenic, adipogenic, and chondrogenic directions and have a similar profile of expression of surface antigens [12,37].

Most of the research in this field has been done on cells isolated from the human limbus. However, before clinical trials of various approaches based on L-MSCs in humans, it is important to test them on model animals. In ophthalmic studies, rabbits are suitable model animals because of the similar eye organization with humans and their size, which makes it easier to work with. Thus, the study of rabbit L-MSCs is an essential step for the development of cell-based technologies in corneal research. These cells could be useful for the development of therapy of the ocular surface, as well as for the study of fundamental biological issues such as cell proliferation and migration in tissues, cell plasticity, and differentiation. Bray et al. compared rabbit BM-MSCs and L-MSCs to develop an animal model of transplantation and showed the low immunogenicity of this culture, which is essential for cell biotechnology [22].

In this work, we obtained a culture of rabbit L-MSCs, as well as L-MSCs-EGFP, which stably expresses a green fluorescent protein. It was shown that both cultures have a high proliferative potential in vitro. We demonstrated that rabbit L-MSCs and L-MSCs-EGFP, as well as rabbit BM-MSCs, express surface antigens CD44, CD90, CD 105, and do not express hematopoietic markers, CD34 and CD45. Expression of CD73 was absent in all cell types, which may be due to the low specificity of antibodies to rabbit antigens. Other authors also highlight the low specificity of antibodies to rabbit antigens [22,38]. These results were consistent with the previous data [22]. We also showed that L-MSCs-EGFP have multilineage differentiation potential and can differentiate into adipocytes, chondrocytes, and osteocytes. Earlier, the cell plasticity of human L-MSCs and their ability to transdifferentiate into various cell types, including neurons, corneal cells, osteoblasts, chondrocytes, adipocytes, cardiomyocytes, hepatocytes, and pancreatic islet cells in vitro has been shown by Dravida et al. [39].

The interaction between L-MSCs and LESCs was shown both in vivo and in vitro [33]. This connection occurs through the pores in the basal membrane and is present only in the limbus area. The basal limbal epithelial stem cells form invaginations through the basal membrane to connect with the underlying matrix, which suggests that stem cells of the limbal epithelium maintain close contact with limbal cells of the stroma [33]. This feature of L-MSCs has been demonstrated in vitro by some authors [40,41]. However, the exact function of L-MSCs in vivo is still a controversial issue.

We observed the presence of stem cell markers ABCG2, ABCB5, PAX6, and p63a in the obtained rabbit L-MSCs and L-MSCs-EGFP populations. These markers are used to characterize LESCs and their presence described in human L-MSCs [9,10,13,42]. We also showed the high level of expression of ALDH3A1 that has been described for corneal epithelial cells and stromal keratocytes [43]. Expression of α-smooth muscle actin (α-SMA) was shown only in some cells within the population. Also, we discovered several cytokeratins (3/12, 15, 19) in L-MSCs and L-MSCs-EGFP, which are usually used in the characterization of corneal epithelial cells [3,44]. Although in previous studies with L-MSCs, the absence of CK3/12 in human cells was shown [12], we demonstrated that in rabbit L-MSCs cultured on plastic, this pair of cytokeratins form fibrillar structures. The presence of epithelial markers such as cytokeratins may indicate their ability to differentiate into epithelial cells under suitable conditions.

We discovered that during cultivation within 14 days in air-lifting conditions in CnT30 medium, the L-MSCs-EGFP acquired epithelial morphology, actin filaments become more cortical, the pattern of expression of CK 15, E-cadherin (epithelial markers), and Vimentin (mesenchymal marker) changed. The fibril structures of CK15 were not detected during epithelial differentiation of L-MSCs-EGFP, but in some cells, a well-defined CK15-ring was formed around the nucleus. A similar pattern for CK 15 expression was previously shown for primary culture of LESCs [45]. Also, an increase in SNAI2 (SLUG) expression by 3.8 times and a decrease in TWIST1 expression in cells cultured in inserts were shown. SLUG and TWIST are described as transcription factors capable of inducing epithelial-mesenchymal transition (EMT) [46]. EMT is a reversible cell process where epithelial cells acquire a mesenchymal phenotype. This is an essential physiological process during embryogenesis, histogenesis, organogenesis, wound healing, and cancer. Previously, it was shown that during this transition, epithelial cells lose intercellular contacts, including E-cadherins, and become more mobile. They also have an increase in the number of N-cadherins and Vimentin—markers of mesenchymal cells. Activation of the SNAIL/SLUG and TWIST suppresses the expression of E-cadherins and induces epithelial-mesenchymal transition [47,48]. Thus, during the reverse mesenchymal-epithelial transition (MET), we expected to see a decrease in both factors. But only TWIST expression was decreased compared to control. L-MSCs-EGFP expressed both epithelial and mesenchymal markers, such intermediate stage between fully-epithelial and fully-mesenchymal states has been described as hybrid E/M state [47], and the balance between EMT and MET regulates cell plasticity [47,49,50].

The possibility of epithelial transdifferentiation of MSCs of various origins has been studied by other authors [14,15,16,18,19]. Some studies have shown the effectiveness of the usage of MSCs cells for the restoration of the corneal epithelium in vivo in model animals [15,16,18] and during the clinical study in humans [8]. But there was no direct evidence of MSCs transdifferentiation into the corneal epithelium in vivo. Nevertheless, Arnhold et al., using labeled GFP MSCs were shown that the labeled cells were integrated into the retinal pigment epithelium and showed the typical hexagonal morphology of retinal pigment epithelial cells [51]. Therefore, L-MSCs also may be a reliable source for epithelial progenitor cells.

During the development of tissue-engineered constructs, special attention is paid to the biocompatibility of the scaffold and the cell component. Even though transparent scaffolds are being developed for ophthalmology, the amniotic membrane and various hydrogels with low transparency are still used as scaffolds. This may interfere with lifetime observation of cell viability and morphology during the development of cell products. Using L-MSCs-EGFP, we showed the difference in the morphology of cells cultured on different scaffolds with low transparency.

Thereby, L-MSCs-EGFP can become a convenient tool for understanding the processes occurring during the treatment using cell-based technologies of various corneal pathologies, including LSCD. Further in vivo studies, including the use of L-MSCs-EGFP, may provide a deeper understanding of the processes of epithelial restoration during MSCs-based therapy.

## Figures and Tables

**Table 1:** Antibodies used in flow cytometry assay.

Antigen	Dilution	Manufacturer, Country	Reactivity
CD34 (GTX75414)	1:100	Genetex, Irvine, CA, USA	Human, Mouse, Rabbit
CD44 (12-0441-82)	1:150	eBioscience, San Diego, CA, USA	Human, Mouse
CD45 (IM1834U)	1:10	Beckman Coulter, Brea, CA, USA	Human
CD73 (550257)	1:30	BD Pharmingen, San Diego, CA, USA	Human
CD90 (555596)	1:100	BD Pharmingen, San Diego, CA, USA	Human
CD105 (560839)	1:200	BD Pharmingen, San Diego, CA, USA	Human
Iso PE (554680)	1:400	BD Pharmingen, San Diego, CA, USA	-

**Table 2:** Antibodies used in immunofluorescence experiments.

Antigen	ICC Dilution	Manufacturer, Country
CK3/12 (ab68260)	1/200	Abcam, Cambridge, UK
CK5 (VP-C400)	1/50	Vector, Burlingame, CA, USA
CK15 (MA5-15567)	1/500	Thermo Fisher Scientific, Waltham, MA, USA
CK19 (ab7754)	1/200	Abcam, Cambridge, UK
P63α (4892S)	1/100	Cell Signalling Technology, Danvers, MA, USA
PAX6 (60433)	1/100	Santa Cruz Biotechnology, Dallas, TX, USA
ALDH3A1 (ab76976)	1/200	Abcam, Cambridge, UK
ABCG2 (ab3380)	1/200	Abcam, Cambridge, UK
αSMA (ab7817)	1/200	Abcam, Cambridge, UK
E-cadherin (ab76066)	1/200	Abcam, Cambridge, UK
Vimentin (ab8978)	1/200	Abcam, Cambridge, UK
Anti-Mouse IgG H&L (ab150114)	1/500	Abcam, Cambridge, UK
Anti-Rabbit IgG H&L (ab150083)	1/500	Abcam, Cambridge, UK

**Table 3:** Primers used in RT-qPCR assay.

Primer Name	Sequence 5′-3′	T °C
ACTA2-For	GTTACTACTGCTGAGCGTGAG	60 °C
ACTA2-Rev	CAGGCAACTCGTAACTCTTC	60 °C
HPRT1-For	CTGGCGTCGTGATTAGTGATGA	60 °C
HPRT1-Rev	ACGTTCAGTCCTGTCCATAATT	60 °C
SNAI1-For	CTCTTTCCTCGTCAGGAAGC	60 °C
SNAI1-Rev	GGCTGCTGGAAGGTAAACTC	60 °C
TWIST1-For	AGCAGGGCCGGAGACCTAGAT	60 °C
TWIST1-Rev	GCCCCACGCCCTGTTTCTTTGA	60 °C

**Table 4:** Surface antigen profile of LSCs and LSCs-EGFP. The ratio of the number of cells expressing the antigen to the total number of cultured cells, %.

Antigen	BM-MSCs	L-MSCs	L-MSCs-EGFP
CD44	97.71%	91.34%	73.84%
CD73	2.11%	2.41%	3.73%
CD90	67.64%	92.86%	74.07%
CD105	88.97%	52.01%	36.59%
CD34	6.39%	4.97%	6.33%
CD45	2.27%	3.30%	3.07%
